# Purification and Product Characterization of Lipoxygenase from Opium Poppy Cultures (*Papaver somniferum* L.)

**DOI:** 10.3390/molecules24234268

**Published:** 2019-11-23

**Authors:** Ivana Holková, Drahomíra Rauová, Michaela Mergová, Lýdia Bezáková, Peter Mikuš

**Affiliations:** 1 Department of Cell and Molecular Biology of Drugs, Faculty of Pharmacy, Comenius University in Bratislava, Kalinčiakova 8, 832 32 Bratislava, Slovakia; mergova1@uniba.sk (M.M.); bezakova@fpharm.uniba.sk (L.B.); 2 Department of Pharmaceutical Analysis and Nuclear Pharmacy, Faculty of Pharmacy, Comenius University in Bratislava, Odbojárov 10, 832 32 Bratislava, Slovakia; rauova.drahomira@fpharm.uniba.sk (D.R.); mikus@fpharm.uniba.sk (P.M.); 3 Toxicological and Antidoping Center, Faculty of Pharmacy, Comenius University in Bratislava, Odbojárov 10, 832 32 Bratislava, Slovakia

**Keywords:** *Papaver somniferum* L., lipoxygenase, purification, lipoxygenase products, positional specificity, HPLC analysis

## Abstract

Opium poppy (*Papaver somniferum* L.) is an ancient medicinal plant producing pharmaceutically important benzylisoquinoline alkaloids. In the present work we focused on the study of enzyme lipoxygenase (LOX, EC 1.13.11.12) from opium poppy cultures. LOX is involved in lipid peroxidation and lipoxygenase oxidation products of polyunsaturated fatty acids have a significant role in regulation of growth, development and plant defense responses to biotic or abiotic stress. The purpose of this study was to isolate and characterize LOX enzyme from opium poppy callus cultures. LOX was purified by ammonium sulfate precipitation and then followed by hydrophobic chromatography using Phenyl-Sepharose CL-4B and hydroxyapatite chromatography using HA Ultrogel sorbent. Sodium dodecyl sulphate-polyacrylamide gel electrophoresis (SDS-PAGE) analysis and immunoblotting revealed that LOX from opium poppy cultures was a single monomeric protein showing the relative molecular weight of 83 kDa. To investigate the positional specificity of the LOX reaction, purified LOX was incubated with linoleic acid and the products were analyzed by high-performance liquid chromatography in two steps, firstly with reverse phase (120-5 Nucleosil C18 column) and secondly with normal phase (Zorbax Rx-SIL column). LOX converted linoleic acid primarily to 13-hydroperoxy-(9*Z*,11*E*)-octadecadienoic acids (78%) and to a lesser extent 9-hydroperoxy-(10*E*,12*Z*)-octadecadienoic acids (22%). Characterization of LOX from opium poppy cultures provided valuable information in understanding LOX involvement in regulation of signaling pathways leading to biosynthesis of secondary metabolites with significant biological activity.

## 1. Introduction

Opium poppy, *Papaver somniferum* L., is one of the world’s oldest medicinal plants producing valuable benzylisoquinoline alkaloids (BIAs). It remains the only commercial source for the narcotic analgesics; morphine, codeine, and semi-synthetic derivatives such as oxycodone and naltrexone [1]. The plant also produces other pharmaceutically important BIAs such as the muscle relaxant papaverine, the antimicrobial agents sanguinarine and berberine, and the antitussive and potential anticancer drug noscapine [2,3]. The biosynthesis of morphine has only been documented in a few plant species restricted to the Papaveraceae family [1]. The content of morphine alkaloids or benzo[*c*]phenanthridine alkaloid sanguinarine, with anti-microbial and potential anti-cancer properties, has made opium poppy one of the most valuable plants in the pharmaceutical industry.

Lipoxygenases (LOXs, linoleate:oxygen oxidoreductases, EC 1.13.11.12) belong to a class of fatty acid dioxygenases occurring both in the plant and animal kingdom. LOX enzymes have also been identified in coral, moss, green microalga, fungi, and bacteria [4,5,6,7,8]. LOXs are non-heme iron containing enzymes, that catalyze the regiospecific oxygenation of polyunsaturated fatty acids with one or more cis,cis-1,4-pentadiene system of double bonds to form conjugated hydroperoxy fatty acids. Linoleic (LA, 18:2) and linolenic acids (LeA, 18:3) are known to be the best substrates for lipoxygenases in plants [9,10]. Arachidonic acid (AA, 20:4) is a preferred substrate for animal LOX enzymes. Animal LOXs have been studied extensively. LOX products in animals are involved in cellular homeostasis, proliferation, and differentiation, and also in pathophysiological processes such as inflammation and cancer [11]. However, during recent years analysis of different plant LOX isoenzymes revealed new knowledge about the LOXs’ structure, catalytic mechanism, regiospecificities, and function also in plants.

The nomenclature of plant LOXs is based on their primary structure and on the positional specificity of linoleic acid oxygenation. Plant LOXs are classified into type I and type II based on sequence similarity, type one LOXs without a transit peptide and a high (>75%) sequence similarity, and type two LOXs with a plastidic transit peptide sequence and a moderate sequence similarity to other LOXs (>35%). Plant LOXs can be further classified into two subfamilies of 9-LOXs and 13-LOXs, that oxygenate fatty acids at the ninth or thirteenth carbon atom, respectively. Until now, all characterized type two LOXs have been shown to exhibit 13-LOX activity. Type one LOXs consist of both 9- and 13-LOXs [10]. Most LOX isoenzymes catalyze the formation of one particular regiospecific isomer. However, several LOX enzymes with dual positional specificity producing both isomers have been characterized as well [12,13,14]. In cells, plant LOXs are mainly soluble, cytoplasmic enzymes, but there is increasing evidence that several isoenzymes are associated with particulate fractions e.g., lipid bodies, vacuoles, or chloroplasts [15].

**Figure 1:**
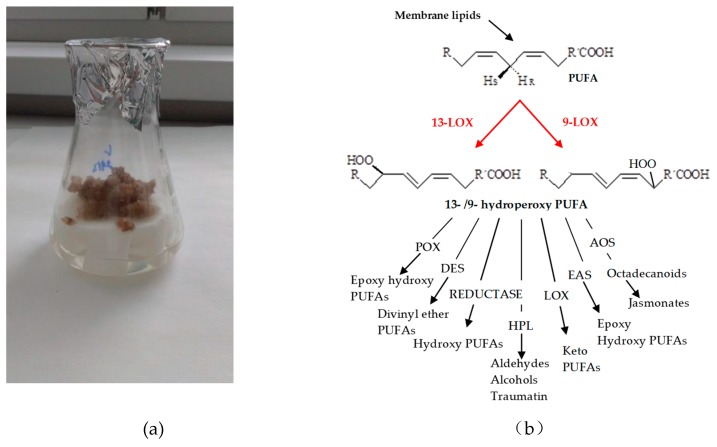
(**a**) Opium poppy (*Papaver somniferum* L.) callus culture; (**b**) The lipoxygenase-catalyzed reaction of polyunsaturated fatty acid (PUFA) into 13-/9-hydroperoxide of PUFA and the scheme of lipoxygenase pathway branches. LOX – lipoxygenase, AOS – allene oxide synthase, DES – divinyl ether synthase, HPL – hydroperoxide lyase, PXG – peroxygenase, EAS – epoxy alcohol synthase.

LOX is the first and a key enzyme in the so-called lipoxygenase (octadecanoid) pathway. Stimulation of the lipoxygenase pathway induces a cascade of reactions leading to the formation of numerous metabolites. The products of the LOX reaction are highly reactive and they are immediately degraded by the activity of other enzymes of LOX pathway branches, including allene oxide synthase (AOS), divinyl ether synthase (DES), hydroperoxide lyase (HPL), peroxygenase (PXG), or epoxy alcohol synthase (EAS) [9]. The scheme of LOX pathway branches are shown in Figure 1b. The final products of lipoxygenase pathway are collectively named oxylipins including the phytohormone jasmonic acid (JA), hydroxy-, oxo-, or keto-fatty acid derivatives, divinyl ethers, or volatile aldehydes. Plant oxylipins have been found to occur as free oxylipins or bound to other molecules such as lipids, glutathione, and amino acids in the form of esters or conjugates [9,15,16]. These products have a role in plant growth, development, and in the defense responses to environmental stress and the defense against microbe and herbivore attack. Several of the volatile products, like jasmonic acid and short-chain aldehydes, have a function in plant-plant communication, or even as bactericidal agents [9,17,18,19,20,21].

The transcriptional regulation of the BIAs pathway in opium poppy during stress response was studied by Mishra et al. [22]. It has been shown that the production of BIAs in poppy plants is stimulated by exposure of the plants to exogenous stresses, such as wounding. The authors Jablonická et al. [23] showed that pharmacological interference with phospholipid signaling pathway caused changes in the secondary metabolism of BIAs in opium poppy (*Papaver somniferum* L.). Our previous study demonstrated the effects of various elicitors on lipoxygenase activity in opium poppy cultures [24] and cultures of California poppy (*Eschscholtzia californica* Cham.) [25]. Our previous research also showed a marked increase in the accumulation of sanguinarine metabolite after elicitation (exogenous addition of stressors) of opium poppy cultures, and the potent LOX inhibitor caused a substantial decrease in sanguinarine production [24]. In vitro cultures provide a convenient year-round model system for study signaling pathways and regulation of pathways for secondary metabolism which could provide the basis for commercial production of desired and medicinally important secondary metabolites [26]. Knowledge of biosynthetic pathways and regulation of BIAs biosynthesis in opium poppy is a key area for future research [1]. Considering the importance of LOX in signaling processes and possible regulation of alkaloid biosynthesis we focused on purification of LOX from opium poppy cultures (Figure 1a).

The main goal of the present work was to isolate and purify the LOX enzyme from opium poppy cultures (*Papaver somniferum* L.) and to determine LOX reaction products by the high-performance liquid chromatography (HPLC) method. LOX was purified and characterized for the first time with the aim of further investigation of its role in signaling processes and alkaloid biosynthesis in opium poppy. The study of signaling pathways and their regulation is an important step in the further secondary metabolites engineering.

## 2. Results and Discussion

### 2.1. Purification and Characterization of LOX from Opium Poppy Cultures

**Figure 2:**
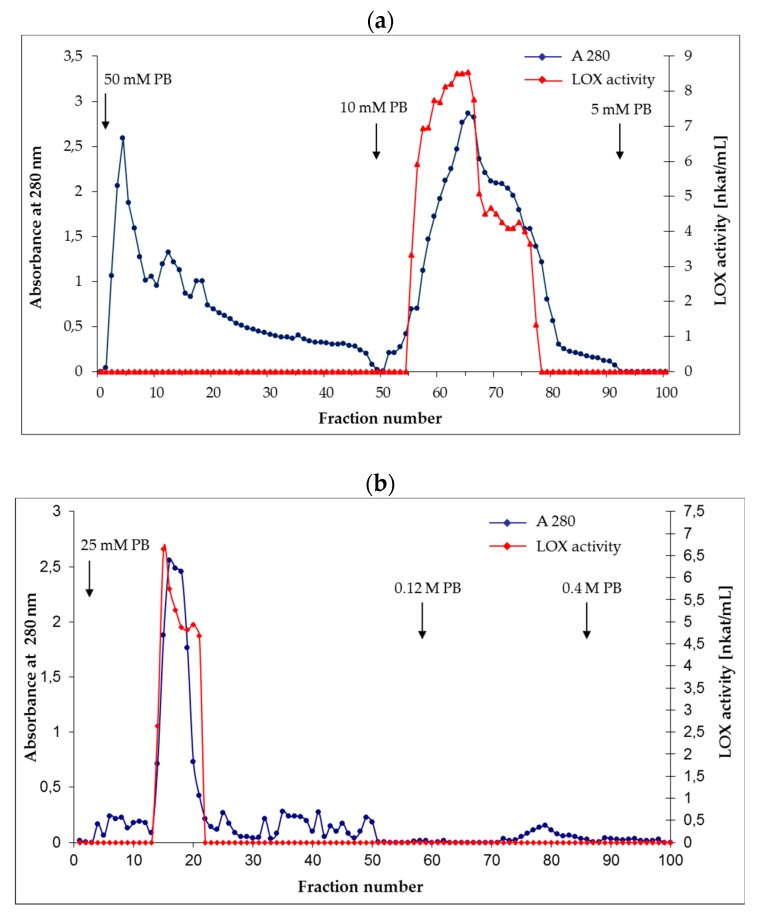
Purification of LOX from opium poppy cultures. (**a**) Elution profile of LOX from opium poppy cultures on a Phenyl-Sepharose CL-4B column. The column was equilibrated with 50 mM phosphate buffer pH 7.0 with 1 mM ammonium sulphate and eluted with 10 mM and 5 mM phosphate buffer pH 6.5. (**b**) Purification on a HA Ultrogel column. The column was equilibrated with 25 mM phosphate buffer pH 6.0 and eluted stepwise with 0.025 M, 0.12 M, and 0.4 M phosphate buffer pH 6.0. Proteins were determined at 280 nm and LOX activity at 234 nm. The arrows indicate the point at which elution buffers were changed. PB – potassium phosphate buffer.

LOX enzyme was purified from opium poppy (*Papaver somniferum* L.) callus cultures using several purification steps. The detailed description of LOX purification is shown in Table 1. First, the plant material was fractionated into a 100,000× *g* soluble fraction and a membrane fraction. The major LOX activity was found in the soluble fraction. For further purification, the proteins in the supernatant were precipitated with ammonium sulphate to 60% saturation and loaded on a Phenyl-Sepharose CL-4B column. The elution profile is shown in Figure 2a. During hydrophobic chromatography a broad single peak of LOX protein was eluted with purification fold of 14.1 and a specific activity of 198.9 nkat/mg. Fractions containing LOX activity were further applied to a HA Ultrogel column. The elution profile is shown in Figure 2b. The specific activity of purified LOX from opium poppy cultures reached 334 nkat/mg. An overall 24-fold purification was achieved (Table 1). The activity of LOX was determined using the optimal pH for this enzyme and linoleic acid as a substrate. It was found to be pH 6.5 in our previous work [24].

Earlier we reported purification and characterization of LOX from germinating seedlings of opium poppy [27] and California poppy [28] and characterization of LOX isoenzyme from chloroplasts of opium poppy leaves [29]. To date, there have been no reports available about purification of LOX from opium poppy cultures. During the past few decades, many LOX isoenzymes were identified from different plant species and their enzymatic properties were determined. Results of our purification procedures correspond with the results obtained by other authors [30]. The purification of LOX enzyme from mung bean by specific operation steps, which included chromatographic methods such as gel filtration, ion exchange chromatography, and chromatofocusing led to the change of enzyme activity, so that 27-fold of purification was obtained [30]. Two isoenzymes of LOX (LOX 1 and LOX 2) were purified from pearl millet mature grains using ammonium sulphate fractionation, gel filtration chromatography, and ion exchange chromatography with a purification fold of 56 for LOX 1 and 40 for LOX 2 [31]. The authors Lorenzi et al. [32] obtained a 65-fold purification of LOX from olives using differential centrifugation and hydrophobic chromatography. LOX activity was also investigated in olive callus cultures, and the most prominent activity was found to be soluble but significant activities were also detected in the plastid fraction [33]. Two LOX isoforms from olive callus cultures were separated and purified by salt precipitation and ion-exchange chromatography on a DEAE Sephadex A50 with 48 and 55 purification factors [34].

**Figure 3:**
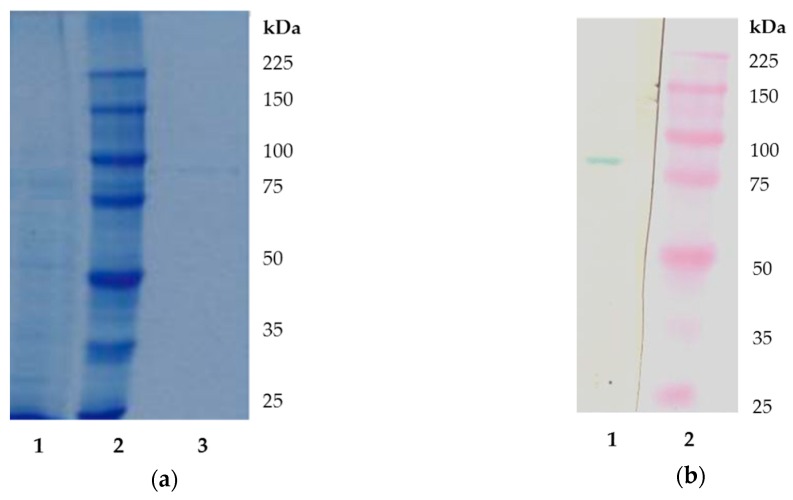
Sodium dodecyl sulphate-polyacrylamide gel electrophoresis (SDS-PAGE) and Western blot analysis of purified LOX from opium poppy cultures (**a**) SDS-PAGE polyacrylamide gel (8%) of purified LOX from opium poppy cultures; Lane one: Purified LOX after HA Ultrogel, Lane two: Marker proteins (25–225 kDa), and Lane three: Commercial soybean LOX (Sigma). (**b**) Western blot analysis of a gel. Lane one: Purified LOX after HA Ultrogel and Lane two: Marker proteins (25–225 kDa).

Analysis of the purified protein by sodium dodecyl sulphate-polyacrylamide gel electrophoresis (SDS-PAGE) in 8% polyacrylamide gel (Figure 3a) and subsequent immunoblotting with anti-soybean LOX antibodies demonstrated that LOX from opium poppy cultures was a single monomeric protein (Figure 3b). Using immunoblotting, one intense band was identified showing the relative molecular weight (Mr) of 83 kDa (Figure 3b, lane one). As a comparison, the sample of commercial soybean LOX (97 kDa) was analyzed (Figure 3a, lane three). The relative molecular weight of purified LOX was similar to previously published data for several plant LOXs such as LOX 1 and LOX 2 isoforms isolated from pearl millet mature grains (Mr of approximately 85 and 79 kDa), LOX from banana leaves (85 kDa), or common bean etiolated hypocotyls (86.7 kDa) as reported [31,35,36]. LOX purified from seedlings of opium poppy had the Mr 78 kDa [27] and California poppy LOX had the Mr 85 kDa [28]. LOX isoenzyme from chloroplasts of opium poppy leaves had higher Mr 92 kDa because of the presence of transit signal sequence [29]. Two LOX isoenzymes characterized from olive callus cultures had molecular masses of around 95 kDa and were found to be associated with plastid membrane fraction [33,34]. The LOX enzyme preparation was used for further characterization of LOX reaction products using HPLC method in two steps began with reverse phase and followed by normal phase.

### 2.2. HPLC Analysis of LOX Reaction Products

Two LOX subfamilies are recognized with respect to their positional specificity, the 9-LOXs and 13-LOXs. The enzyme is described as 13-LOX when the product formed is 13-hydroperoxy-9(*Z)*,11(*E*)-octadecadienoic acid (13-HPODE) and 9-LOX when the 9-hydroperoxy-10(*E*),12(*Z*)-octadecadienoic acid (9-HPODE) is predominantly produced [10]. However, LOX enzymes are not perfectly specific and LOXs that produce more than 10% of the alternative regio-isomer are called dual positional specific LOXs [37].

**Figure 4:**
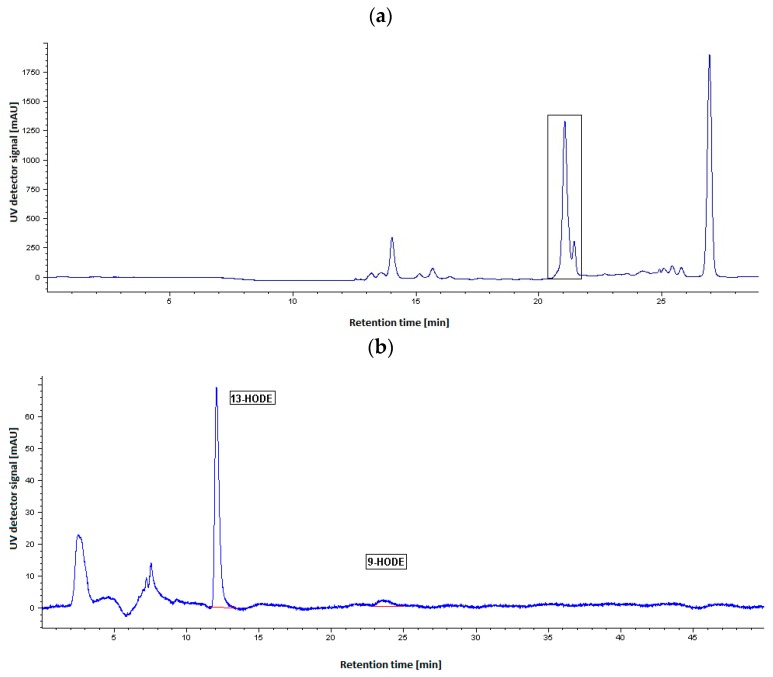
HPLC analysis of incubation products of purified LOX enzyme and linoleic (LA) substrate. (**a**) Chromatographic profile of the preparative reversed-phase high-performance liquid chromatography (RP-HPLC). The marked peak represents an isolated fraction (absorbing at 234 nm) used for the subsequent NP-HPLC separation of hydroxy-octadecadienoic acids (HODEs). The eluate of the isolated fraction was collected in the interval of 21 min. (**b**) Chromatographic profile of the NP-HPLC separation of 13-/9-HODE products. The elution times of 13-HODE and 9-HODE were 12 min and 23.5 min, respectively. The unidentified peaks in NP-HPLC profile could be structurally related compounds such as fatty acids originating from the plant matrix (based on their similar elution and UV absorbance properties).

To investigate the positional specificity of purified LOX from opium poppy cultures, HPLC analysis with properly selected stationary phases was performed. Reaction specificity of LOX was determined after its incubation with linoleic acid as a substrate using a preparative separation of the obtained sample by the reversed-phase high-performance liquid chromatography (RP-HPLC) followed by the normal-phase HPLC technique (NP-HPLC). LOX products, hydroperoxy fatty acids, formed during the reaction were reduced to their corresponding hydroxy fatty acids, extracted, and then separated from the incubated sample matrix. The RP-HPLC effectively removed a majority of the interfering residual sample matrix constituents present in the incubation mixture (e.g., LA products as well as other possible structurally related compounds originating from the plant matrix). The results are depicted in Figure 4a. The eluate containing hydroxy fatty acids (the marked peak absorbing at 234 nm) was collected and analyzed by NP-HPLC.

NP-HPLC was used as an analytical step to complete the separation of products that coeluted in a RP-HPLC column. 13-hydroxy-octadecadienoic acid (HODE) eluted a bit earlier than 9-HODE (Figure 4b). For a positive identification, the retention times of the reaction products of poppy LOX were compared with authentic standards of 13(*S*)-hydroxy-(9*Z*,11*E*)-octadecadienoic acid and 9(*S*)-hydroxy-(10*E*,12*Z*)-octadecadienoic acid. Quantitation of these products was achieved from the calibration curves using 13-HODE or 9-HODE standards.

The HPLC analysis of the products of purified LOX from opium poppy cultures showed a dual positional specificity of the enzyme (see chromatographic profile in Figure 4b). The major reaction product was 13-HODE (78%), while 9-HODE was formed at 22%, when LA was used as a substrate at pH 6.5. The concentration of 13-HODE in the incubation mixture was calculated to be 3.34 ± 0.1 μg/mL and 9-HODE was 0.94 ± 0.06 μg/mL. Our findings are consistent with previous results obtained for LOXs purified and characterized from other Papaveraceaes such us opium poppy seedlings [27], California poppy LOX [28] and chloroplast poppy LOX [29]. Other plant LOXs with dual positional specificity, classified as nontraditional LOX enzymes, were isolated and characterized in maize seedlings [12], in developing rice seeds [13], in apple fruit [37], or in tea plant [38]. LOXs from olive fruit [32], pea [39], barley [40], or cucumber seedlings [41] were classified as 13-LOX. LOXs from banana leaf [36], tomato fruits [42], several LOXs isoenzymes from pepper [43], or tea plant [38] were described as 9-LOX and formed 9-HPODE as the major product.

Hydroperoxides, the primary products of LOX, are transformed into various biologically active metabolites. Different LOX-isoforms provide various pools of biologically active oxilipins from hydroperoxy polyunsaturated fatty acids [44] and may have different effects on plant physiology [10]. The 13-LOX derived oxylipins such as JA and its precursor 12-oxo-phytodienoic acid (OPDA) or green leaf volatiles (GLVs) have been well characterized for their significant roles during development and in direct or indirect plant defense responses. In indirect plant defense, GLVs play a pivotal role in the attraction of natural enemies of the herbivores. In the contrary to 9-LOX activity, 13-LOX activity leads to the biosynthesis of JA [9]. 9-LOXs are mainly involved in the functions such as plant-pathogen interactions, regulation of plant growth, tuber development, or in the formation of flavor compounds [9,36]. The 9- and 13-LOXs pathways were shown to be spatially separated. The 13-LOX type one proteins are preferentially found in the cytoplasm, while the 13-LOX type two ones are located in chloroplasts. The 9-LOX pathway occurs in cytoplasm. 9-HPODE production is associated primarily with the cytoplasmic membrane. It was suggested that soluble LOXs may be transferred to membranes, where they may attack more easily their substrates—polyunsaturated fatty acids, linked to phospholipids, or liberated by phospholipases. Cho et al. [14] provided evidences for calcium-mediated translocation of dual positional specific maize LOX without chloroplast targeting sequence (type one LOX) from cytoplasm with chloroplast membranes in plants. The positional specificity of LOXs is an important enzyme property and may help to predict function of specific LOX isoenzymes. The dual positional specific LOXs provide hydroperoxides for both pathways and can produce a greater range of final products in LOX pathway than strictly 13-LOX or 9-LOX. Kim et al. [12] reported the expression of a dual positional specific maize LOX in response to wounding or methyl jasmonate. LOX from developing rice seeds with dual positional specificity responds to wounding and insect attack [13]. Zhu et al. [38] identified several LOX genes from the tea plant (*Camellia sinensis*). CsLOX1 and CsLOX2 isoenzymes exhibited dual positional specificity. CsLOX2 was upregulated after attack by the insect, while CsLOX1 was induced after infection with the pathogen and JA. The barley LOX2.2 gene was studied by Bachmann et al. [40]. Assays of the recombinant enzyme with LA showed that the products were 13-HPODE. Losvik et al. [21] studied overexpression and down-regulation of the lipoxygenase gene LOX2.2 in barley (*Hordeum vulgare* L.) and confirmed that LOX2.2 had a role in the activation of JA-mediated defense responses. This enzyme was characterized as a chloroplastic 13-LOX. A rice LOX, encoded by OsLOX1 gene, was localized to chloroplasts. It has dual positional specificity, as it releases both C-9 and C-13 oxidized products in a 4:3 ratio and responds to wounding and insect attack [13]. Williams et al. [33] investigated LOX activity in olive callus cultures and found the evidence of several LOX isoforms involved in the growth cycle of olive callus. Both isoforms characterized from olive callus cultures preferentially formed the 13-hydroperoxy products [34]. LOX from opium poppy cultures had dual positional specificity, but it preferentially produced 13-HPODE compounds. Based on the HPLC characterization of opium poppy LOX predominantly as 13-LOX, we assume that this enzyme could be involved in growth of callus cultures and in the induction of defense responses. Such conclusion is consistent with the findings of the authors cited above in the text.

## 3. Materials and Methods

### 3.1. Plant Material

Callus cultures of *Papaver somniferum* L. cv. Lazur were established at the Department of Cell and Molecular Biology of Drugs, Faculty of Pharmacy, Comenius University in Bratislava according to the reported procedure [45]. Callus cultures were maintained on Murashige and Skoog medium supplemented with 0.1 mg/L kinetin, 2 mg/L α-naphtylacetic acid, and 30 g/L sucrose [46]. The cultures were routinely subcultured at 28 days intervals.

### 3.2. Enzyme Purification

Opium poppy callus cultures (70 g) were homogenized in 125 mL of 25 mM potassium phosphate buffer (pH 6.0) containing 0.5 mM ethylenediaminetetraacetic acid, 1 mM phenylmethyl sulphonyl fluoride, 1 mM cysteine hydrochloride, 10 mM sodium thiosulphate, and 0.4% (w/v) Polyclar AT. The homogenate was filtered through two layers of cheesecloths and centrifuged 15 min at 12,000× *g*. Then the supernatant fluid was centrifuged at 100,000× *g* for 30 min (JS 24.38 rotor, Beckman Coulter, USA), in order to separate the insolubilized membranes (pellet) from the solubilized fraction. Both fractions were assayed for LOX activity. The activity of LOX in the membrane fraction was determined after adding 25 mM potassium phosphate buffer (pH 6.0) containing 0.1% (v/v) Triton X-100. All extraction and purification procedures were performed at 4 °C. The proteins in supernatant fluid were precipitated with ammonium sulphate to 60% (w/v) saturation and centrifuged at 15,000× *g* for 30 min. The concentrated protein sample was dissolved in 3 mL of 50 mM potassium phosphate buffer (pH 7.0) containing 1 M ammonium sulphate and applied to a Phenyl-Sepharose CL-4B column (Ø 1.5 × 15 cm, Sigma-Aldrich, St. Louis, USA) equilibrated with the same buffer. The resin was washed with 50 mM potassium phosphate buffer (pH 7.0) containing 1 M ammonium sulphate, and bound proteins were eluted with 10 mM potassium phosphate buffer (pH 6.5) containing 0.5 mM glutathione and 0.04% (v/v) Tween 20, followed by 5 mM potassium phosphate buffer (pH 6.5) containing 0.5 mM glutathione and 5 mM ethylenediaminetetraacetic acid. Fractions of 2 mL were collected at the flow rate of 1 mL/min. The protein elution profile was measured spectrophotometrically at 280 nm and LOX activity was determined at 234 nm.

Active fractions were pooled, freeze-dried and dissolved in 3 mL of 25 mM potassium phosphate buffer (pH 6.0) and applied to a HA Ultrogel^®^ column (Ø 2 × 15 cm, Sigma-Aldrich, St. Louis, USA) equilibrated with the same buffer and eluted stepwise with 0,025 M, 0.12 M, and 0.4 M potassium phosphate buffer (pH 6.0) at the flow rate of 1 mL/min. Fractions of 2 mL were collected. The eluate was monitored at 280 nm. Each fraction was assayed for LOX activity. Fractions with LOX activity were pooled and concentrated using 50 kDa membrane filter Amicon Ultra Centrifugal Filters Ultracel^®^-50K (Milipore, USA) using centrifugation at 15,000× *g* for 30 min and at 4 °C. The LOX enzyme preparation was then stored at −20 °C until further analysis. 

### 3.3. Measurement of LOX Activity and Protein Determination

The activity of LOX was determined spectrophotometrically at room temperature by measuring the increase of absorbance at 234 nm. UV/VIS Spectrometer (Lambda 35, Perkin Elmer, USA) was used. The substrate—linoleic acid (Sigma-Aldrich, St. Louis, USA) was prepared according to the reported procedure [39]. The reaction mixture contained 920 µL of 100 mM potassium phosphate buffer (pH 6.5), 105 µL of substrate solution (10 mM), and 25 µL of LOX enzyme preparation. The LOX activity was expressed in katals. The protein content was determined according to the Bradford assay using bovine serum albumin (Sigma-Aldrich, St. Louis, USA) as a standard [47].

### 3.4. SDS-PAGE and Immunoblotting

SDS-PAGE was performed in a Mini-PROTEAN^®^ 3 CELL vertical electrophoresis apparatus (Bio-Rad, Richmond, USA) according to the method of Laemmli [48] using 8% polyacrylamide gel. Standard molecular weight markers (10–225 kDa) from Novagen (USA) were used as reference. The gel was stained for proteins using PageBlue^TM^ solution containing Coomassie Brilliant Blue G-250 (Thermo Scientific, Waltham, USA). After electrophoresis, proteins were transferred to the nitrocellulose membrane using Trans-Blot SD Semi-Dry Transfer Cell (Bio Rad, Richmond, USA) according to manufacturer’s instructions. The LOX was detected using immunoblot method with anti-LOX serum. The secondary antibody reaction was carried out using goat anti-rabbit IgG secondary antibody conjugated to peroxidase (Scintila, Czech Republic). Reaction was visualized with 3,3’,5,5’-tetramethylbenzidine (TMB stabilized substrate for horse radish peroxidase, Promega, Madison, USA). Polyclonal anti-LOX serum was prepared against soybean LOX as reported previously [24]. The molecular mass of purified enzyme was estimated by comparing the mobility of LOX protein with the mobility of molecular markers in 8% SDS-polyacrylamide gel. Standard molecular weight markers (10–225 kDa) were used to make a plot of the logarithm of molecular mass versus the relative mobility of protein bands.

### 3.5. HPLC Analysis of LOX Reaction Products

High-performance liquid chromatography analysis of the LOX products was performed on an Agilent Technologies 1050 series HPLC system (Waldbronn, Germany) coupled to an UV detector. The absorbance was recorded at 234 nm. For product analysis, 100 µL of purified LOX enzyme preparation was added to 900 µL of 100 mM potassium phosphate buffer (pH 6.5) and incubated with 10 µL of the substrate (10% methanol solution of LA, v/v). The reaction was allowed to proceed for 30 min at room temperature. Then, it was stopped by acidification with 100 µL of concentrated HCl and hydroperoxides formed were reduced to their corresponding hydroxides with 100 mg of NaBH_4_. Hydroxy-octadecadienoic acids were extracted with diethyl ether (2 times 1 mL) and evaporated to dryness in the nitrogen stream. After removing the organic solvent, the residue was reconstituted in 0.2 mL of mobile phase (methanol/water/acetic acid, 85:15:0.1, v/v/v). Aliquots of 70 µL were analyzed by reverse-phase high-performance liquid chromatography (RP-HPLC) according to Vanko et al. [29].

RP-HPLC was carried out on a column 120-5 Nucleosil C18 (250 × 4 mm, Watrex, Czech Republic) with a gradient system of solvent A (methanol/acetic acid, 99:1, v/v) and solvent B (water). The program of elution was as follows: 10 min with solvent system of 85% A and 15% B at a flow rate of 0.2 mL/min; 12 min with 100% A, flow rate 0.4 mL/min and 5 min with 85% A and 15% B, flow rate 0.4 mL/min. The eluate containing hydroxy fatty acids (peak fraction at 234 nm) was collected and evaporated to dryness under a stream of nitrogen. The residue was dissolved in 150 µL of hexane and aliquots of 50 µL were analyzed by NP-HPLC. The NP-HPLC method was performed on a Zorbax Rx-SIL column (150 × 2.1 mm, 5 µm particle size, Agilent Technologies, Waldbronn, Germany) eluted with a solvent system of hexane/2-propanol/acetic acid (99:1:0.1, v/v/v) at a flow rate of 0.2 mL/min. The absorbance at 234 nm (conjugated diene system of the hydroxy fatty acids) was recorded simultaneously during all chromatographic steps. The identity of products were confirmed by co-chromatography with the authentic standards 9-HODE (5 µg/mL) and 13-HODE (5 µg/mL) purchased from Cayman Pharma (Czech Republic). For the quantification of LOX products by NP-HPLC, calibration curves for 9- and 13-HODE were obtained in the range of 0.84–100 µg/mL and 1.29–100 µg/mL.

## 4. Conclusions

In the present study, LOX from opium poppy callus cultures was purified and characterized for the first time. LOX enzyme purification procedure was innovative with the benefits of its simplicity and reduced operation time which included ammonium sulphate precipitation followed by hydrophobic chromatography and hydroxyapatite chromatography. One isoform of enzyme from cytosolic fraction of callus cultures was partially purified and subsequently analyzed by combination of chromatographic methods with different polarity of stationary phases. The relative molecular weight of purified LOX was estimated to be 83 kDa by immunoblotting. The results indicate that LOX from opium poppy cultures belongs to the group of nontraditional plant LOXs with dual positional specificity. LOX from opium poppy cultures preferentially formed the 13-HPODE (3.34 ± 0.1 μg/mL) and only a lesser extent of 9-HPODE (0.94 ± 0.06 μg/mL) during the reaction of purified LOX with LA substrate. Our findings also suggest that LOX from opium poppy cultures, which is a cytosolic enzyme without a plastidic transit peptide sequence, could be considered as a type one LOX. In our continuing experiments it will be confirmed by LOX expression studies and sequencing of the LOX gene.

LOX from opium poppy cultures characterization could improve scientific knowledge about LOX in plants and our understanding of its involvement in regulation of signaling pathways leading to secondary metabolite biosynthesis. The present study importance is highlighted by the role of secondary metabolites from opium poppy in pharmaceutical industry.

## Figures and Tables

**Table 1:** Purification summary of LOX from opium poppy cultures.

Purification Step	Activity (nkat/mL)	Proteins (mg/mL)	Specific Activity (nkat/mg)	Purification (fold)
Crude extract 100,000× *g* soluble fraction	937.20 789.40	66.55 40.77	14.08 19.36	1.0 1.4
Phenyl-Sepharose CL-4B	208.90	1.05	198.9	14.1
HA Ultrogel	136.94	0.41	334.0	24.0
